# Impact of Site Size on Data Quality in Early Alzheimer’s Disease Clinical Trials

**DOI:** 10.1002/alz.088870

**Published:** 2025-01-09

**Authors:** Alan Kott, Xingmei Wang, Petra Reksoprodjo, Andrei Iacob, David S. Miller

**Affiliations:** ^1^ Signant Health, Prague Czech Republic; ^2^ Signant Health, Blue Bell, PA USA; ^3^ Signant Health, Iasi Romania

## Abstract

**Background:**

Clinical trial sponsors rely on research sites to identify and enroll appropriate study participants and to correctly and reliably assess symptom severity and function over the course of the trial. Low‐recruiting sites represent a large financial and operational burden and may negatively impact trial success either by selecting inappropriate participants and/or high prevalence of data quality issues. We previously reported that >60% of sites in schizophrenia clinical trials recruited ≤5 participants. Here we analyze 3 large dementia trials to assess the proportion of low‐recruiting sites and compare their data quality with the remaining sites.

**Method:**

Data were obtained from 3 large early dementia clinical trials totaling 834 sites. Sites were divided into two groups based on the number of randomized subjects: 1. sites with more than 5 randomized subjects (high‐recruiting sites) and 2. sites with 5 or less randomized subjects (low‐recruiting sites). Data quality issues were defined as administration and scoring errors on relevant scales. These errors were summed per site and then compared by site size using Poisson regression with the site size, study and their interaction as predictors and the number of possible hits as exposure.

**Result:**

71 (8.5%) sites did not randomize a subject, 43 (5.2%) sites randomized one subject, and 377 (45.2%) sites randomized ≤5 subjects. Overall, administration and scoring errors were more frequent at low‐recruiting sites (seen at 41.9% visits) compared to 35.3% at the high‐recruiting sites. 2 studies show significantly higher IRRs, 1.59 (1.46, 1.74) and 1.2 (1.15, 1.25) respectively, while the third study has an IRR close to 1 but not statistically significant.

**Conclusion:**

Our results indicate that low‐recruiting sites (arbitrarily defined as randomizing ≤5 participants) are frequent in large dementia trials. These sites pose considerable cost to sponsors, but more importantly, are more likely to provide questionable data quality. While a single site like this only represents a small risk, in aggregate, they can pose a serious challenge. Clinical trial sponsors should therefore consider strategies to minimize the impact of low‐recruiting sites on study outcomes.

